# Redetermined structure of methyl 3-{4,4-di­fluoro-2-[2-(methoxy­car­bon­yl)­ethyl]-1,3,5,7-tetra­methyl-4-bora-3a,4a-di­aza-*s*-in­da­cen-6-yl}pro­pion­ate

**DOI:** 10.1107/S2414314624008848

**Published:** 2024-09-17

**Authors:** Dieter Schollmeyer, Matthias Jochen, Heiner Detert

**Affiliations:** a University of Mainz, Department of Chemistry, Duesbergweg 10-14, 55099 Mainz, Germany; University of Aberdeen, United Kingdom

**Keywords:** crystal structure, bodipy dye, boron, fluorine,

## Abstract

In the title compound, C_21_H_27_BF_2_N_2_O_4_, a highly fluorescent boron–dipyrromethene dye, the methyl­propionate moieties have different conformations. In the crystal, weak C—H⋯F and C—H⋯O inter­actions link the mol­ecules. Some optical properties are presented.

## Abstract 2

In the title compound, a highly fluorescent boron–dipyrromethene dye, the methyl­propionate moieties have different conformations. In the crystal, weak C—H⋯F and C—H⋯O inter­actions link the mol­ecules.

## Graphical abstract

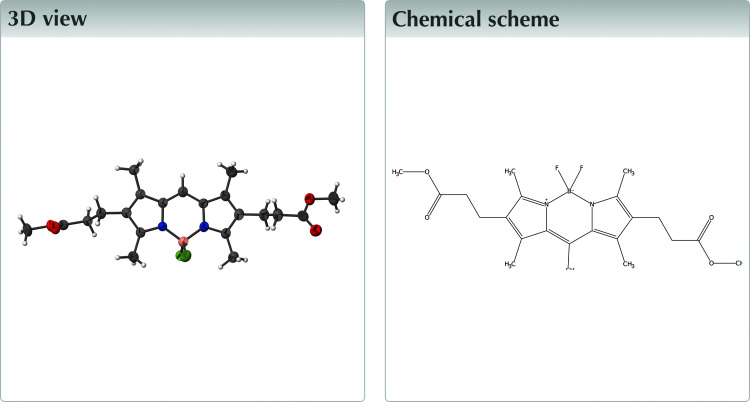

## Structure description

**Figure 1:**
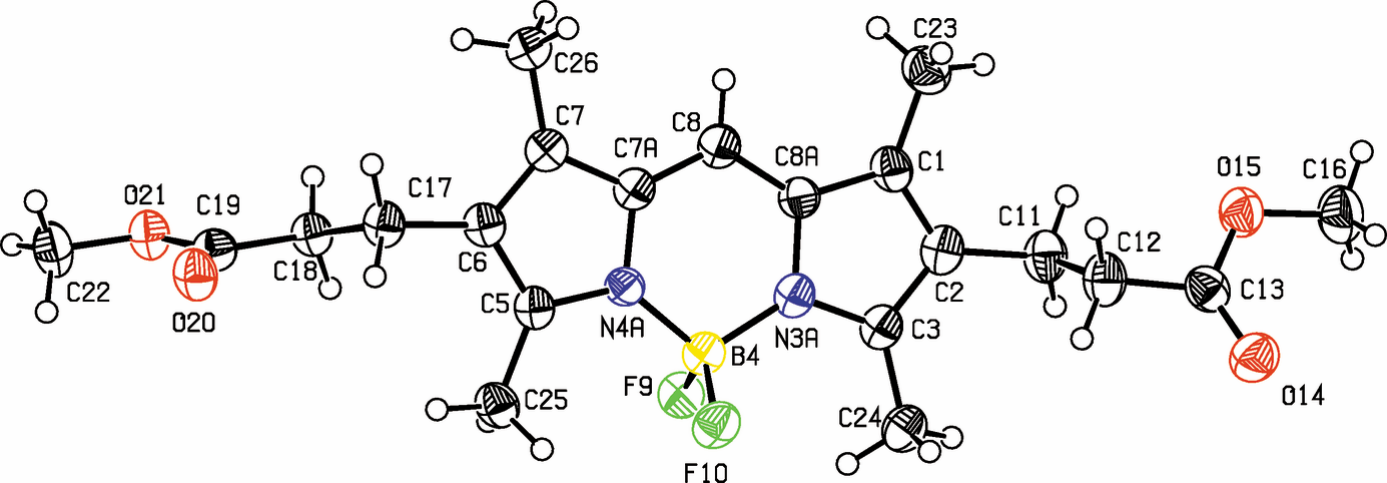
View of compound **I**. Displacement ellipsoids are drawn at the 50% probability level.

Fluorescent dyes are of great inter­est for labeling for analytics in biology and medicine (*e.g.*, Carpenter & Verkman, 2010 ▸; He *et al.*, 2003 ▸; Marfin *et al.*, 2017 ▸; Namkung *et al.*, 2009 ▸). Boron–dipyrromethene dyes (bodipy) show high quantum yields and excellent photostability and 9-aryl-substituted compounds have been the most investigated. The syntheses of these dyes usually consist of the condensation of pyrroles with aldehydes. To synthesize 9*H*-bodipy dyes, orthoformates have been used but here, di­methyl­formamide is the source of the central carbon atom in the title compound, C_21_H_27_BF_2_N_2_O_4_ (**I**) (Fig. 1 ▸).

Despite the high formal symmetry of **I**, the mol­ecule shows no inherent symmetry in its crystalline form. This is due to the methyl­propionate moieties: the C17 branch adopts an all-*anti* conformation lying to one side of the π-system, while the C11 branch has an *s–cis* conformation on the other side of the π-system. The dihedral angles of these units with respect to the central fused-ring system are 84.3 (2) (C17 branch) and 74.6 (2)° (C11 branch). The 2,3,4-tris­ubstitution on the pyrrole rings enlarges the bond angles involving the methyl groups [C6—C5—C25 = 127.7 (4); C6—C7—C26 = 128.3 (4); C2—C1—C23 = 127.9 (4); C2—C3—C24 = 127.9 (4)°]. The near identical B4—N3*A* [1.537 (6) Å] and B4—N4*A* [1.535 (6) Å] bond lengths indicate the expected delocalization of charge (compare the chemical scheme).

**Figure 2:**
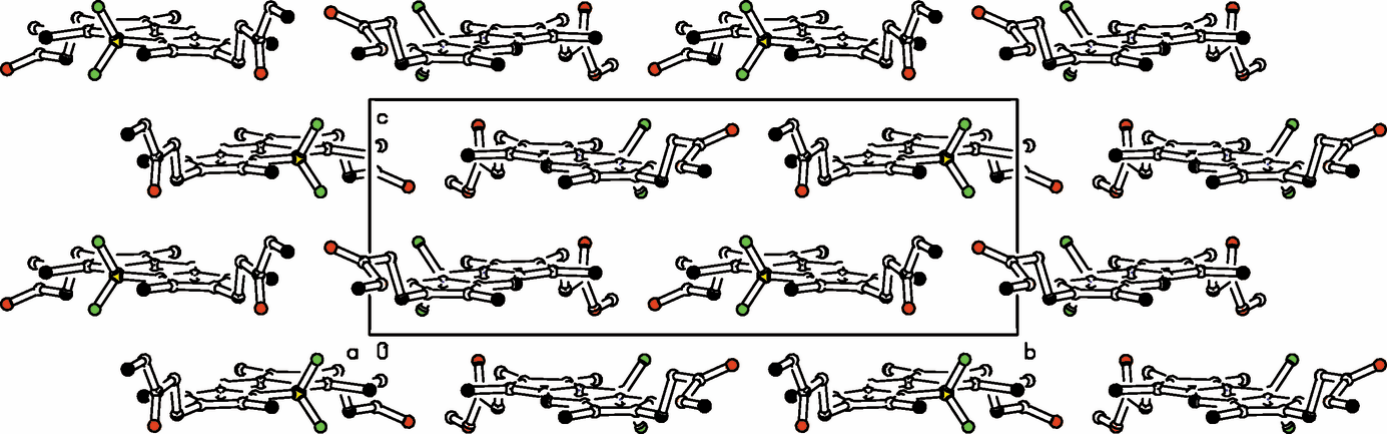
Partial packing diagram. View along the *a* axis.

In the extended structure of **I**, four mol­ecules fill the unit cell, which are arranged in layers lying parallel to the *ac*-plane and weak C—H⋯ and C—H⋯O inter­actions link the mol­ecules ((Fig. 2 ▸), Table 1 ▸). Within the plane a herringbone pattern is formed and a twofold screw axis relates the mol­ecular entities. These crystallographic results confirm recently reported deposited data (Uppal *et al.*, 2020 ▸).

## Synthesis and crystallization

The compound was obtained as a side-product in the condensation of the pyrrole with a formyl­ated cryptand (Jochem *et al.*, 2022 ▸). To the cryptand (97 mg), containing 5% di­methyl­formamide (4.9 mg, 0.066 mmol) in dry chloro­form (10 ml) was added 3,5-dimethyl-4-(meth­oxy­carbon­yl)eth-2-yl­pyrrole (65.0 mg, 0.359 mmol). Then, 10 µl of tri­fluoro­acetic acid was added and stirred for 26 h. Diiso-propyl­ethyl­amine (1 ml) was added followed by di­chloro­dicyano­quinone (54 mg) and stirred for 2 h. Afterwards, BF_3_ diethyl ether solution (40%, 1 ml) was added dropwise and stirred. After complete addition, the mixture slowly turned red–violet and started fluorescing after about one h. After 20 h and addition of water (MilliQ, 20 ml), the organic phase was separated, washed with water and dried over Na_2_CO_3_. Purification *via* column chromatography (SiO_2_/CH_2_Cl_2_) led to the title compound being eluated first: it crystallized from chloro­form/2-propanol as a red solid (12.5 mg) and was recrystallized readily from the mixed solvents of aceto­nitrile and methanol. HR–ESI–MS: found: 421.2106 [*M* + H]^+^, calculated 421.2105 for C_21_H_27_BF_2_N_2_O_4_^+^; ^1^H NMR (400 MHz, CDCl_3_) δ = 6.98 (*s*, 1H), 3.67 (*s*, 6H), 2.71 (*dd*, *J* = 8.6, 6.9 Hz, 4H), 2.50 (*s*, 6H), 2.44 (*dd*, *J* = 8.6, 7.0 Hz, 4H), 2.19 (*s*, 6H). ^13^C NMR (101 MHz, CDCl_3_) δ = 173.25, 155.23, 137.92, 132.64, 128.15, 119.38, 51.86, 34.27, 19.65, 12.80, 9.71. ^19^F NMR (282 MHz, CDCl_3_) δ = −146.29 (*dd*, *J* = 66.4, 33.2 Hz). Optical properties: the title compound has a high of solubility in a broad range of polar solvents but very limited solubility in toluene and alkanes. Bright sunlight led to photochemical decomposition only in very polar media whereas 10^−5^*M* solutions in less polar solvents remained stable. The absorption spectra in CH_2_Cl_2_ shows a peak at 527 nm with emission at 536 nm: increasing solvent polarity provokes bathochromic shifts of max. 3 nm.

## Refinement

Crystal data, data collection and structure refinement details are summarized in Table 2 ▸.

## Supplementary Material

Crystal structure: contains datablock(s) I, global. DOI: 10.1107/S2414314624008848/hb4485sup1.cif

Structure factors: contains datablock(s) I. DOI: 10.1107/S2414314624008848/hb4485Isup2.hkl

CCDC reference: 2263103

Additional supporting information:  crystallographic information; 3D view; checkCIF report

## Figures and Tables

**Table 1:** Hydrogen-bond geometry (Å, °)

*D*—H⋯*A*	*D*—H	H⋯*A*	*D*⋯*A*	*D*—H⋯*A*
C16—H16*A*⋯F9^i^	0.98	2.37	3.324 (7)	163
C18—H18*A*⋯O14^ii^	0.99	2.57	3.268 (6)	128
C23—H23*B*⋯O20^iii^	0.98	2.54	3.470 (7)	158

Symmetry codes: (i) 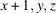; (ii) 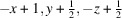; (iii) 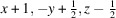.

**Table 2:** Experimental details

Crystal data	
Chemical formula	C_21_H_27_BF_2_N_2_O_4_
*M*_r_	420.25
Crystal system, space group	Monoclinic, *P*2_1_/*c*
Temperature (K)	120
*a*, *b*, *c* (Å)	11.9299 (8), 21.6278 (17), 8.2665 (6)
β (°)	108.251 (6)
*V* (Å^3^)	2025.6 (3)
*Z*	4
Radiation type	Mo *K*α
μ (mm^−1^)	0.11
Crystal size (mm)	0.20 × 0.10 × 0.04

Data collection	
Diffractometer	Stoe *IPDS* 2T
Absorption correction	Integration (*X-RED32*; Stoe & Cie, 2020 ▸)
*T*_min_, *T*_max_	0.985, 0.995
No. of measured, independent and observed [*I* > 2σ(*I*)] reflections	9943, 4863, 2663
*R*_int_	0.052
(sin θ/λ)_max_ (Å^−1^)	0.664

Refinement	
*R*[*F*^2^ > 2σ(*F*^2^)], *wR*(*F*^2^), *S*	0.090, 0.238, 1.09
No. of reflections	4863
No. of parameters	277
H-atom treatment	H-atom parameters constrained
Δρ_max_, Δρ_min_ (e Å^−3^)	0.50, −0.48

Computer programs: *X-AREA WinXpose*, *Recipe* and *Integrate* (Stoe & Cie, 2020 ▸), *SHELXT2014* (Shelxdrick, 2015*a* ▸), *SHELXL2018/3* (Sheldrick, 2015*b* ▸) and *PLATON* (Spek, 2020 ▸).

## full crystallographic data

### Methyl 3-{4,4-difluoro-2-[2-(methoxycarbonyl)ethyl]-1,3,5,7-tetramethyl-4-bora-3a,4a-diaza-s-indacen-6-yl}propionate Crystal data

**Table:**

C_21_H_27_BF_2_N_2_O_4_	*F*(000) = 888
*M**_r_* = 420.25	*D*_x_ = 1.378 Mg m^−^^3^
Monoclinic, *P*2_1_/*c*	Mo *K*α radiation, λ = 0.71073 Å
*a* = 11.9299 (8) Å	Cell parameters from 6884 reflections
*b* = 21.6278 (17) Å	θ = 2.6–28.3°
*c* = 8.2665 (6) Å	µ = 0.11 mm^−^^1^
β = 108.251 (6)°	*T* = 120 K
*V* = 2025.6 (3) Å^3^	Plate, red
*Z* = 4	0.20 × 0.10 × 0.04 mm

### Methyl 3-{4,4-difluoro-2-[2-(methoxycarbonyl)ethyl]-1,3,5,7-tetramethyl-4-bora-3a,4a-diaza-s-indacen-6-yl}propionate Data collection

**Table:**

Stoe IPDS 2T diffractometer	4863 independent reflections
Radiation source: sealed X-ray tube, 12 x 0.4 mm long-fine focus	2663 reflections with *I* > 2σ(*I*)
Detector resolution: 6.67 pixels mm^-1^	*R*_int_ = 0.052
rotation method, ω scans	θ_max_ = 28.2°, θ_min_ = 2.6°
Absorption correction: integration (XRED32; Stoe & Cie, 2020)	*h* = −15→15
*T*_min_ = 0.985, *T*_max_ = 0.995	*k* = −26→28
9943 measured reflections	*l* = −10→10

### Methyl 3-{4,4-difluoro-2-[2-(methoxycarbonyl)ethyl]-1,3,5,7-tetramethyl-4-bora-3a,4a-diaza-s-indacen-6-yl}propionate Refinement

**Table:**

Refinement on *F*^2^	Primary atom site location: dual
Least-squares matrix: full	Hydrogen site location: inferred from neighbouring sites
*R*[*F*^2^ > 2σ(*F*^2^)] = 0.090	H-atom parameters constrained
*wR*(*F*^2^) = 0.238	*w* = 1/[σ^2^(*F*_o_^2^) + (0.0569*P*)^2^ + 5.4328*P*] where *P* = (*F*_o_^2^ + 2*F*_c_^2^)/3
*S* = 1.09	(Δ/σ)_max_ < 0.001
4863 reflections	Δρ_max_ = 0.50 e Å^−^^3^
277 parameters	Δρ_min_ = −0.48 e Å^−^^3^
0 restraints	

### Methyl 3-{4,4-difluoro-2-[2-(methoxycarbonyl)ethyl]-1,3,5,7-tetramethyl-4-bora-3a,4a-diaza-s-indacen-6-yl}propionate Special details

**Table:**

Geometry. All esds (except the esd in the dihedral angle between two l.s. planes) are estimated using the full covariance matrix. The cell esds are taken into account individually in the estimation of esds in distances, angles and torsion angles; correlations between esds in cell parameters are only used when they are defined by crystal symmetry. An approximate (isotropic) treatment of cell esds is used for estimating esds involving l.s. planes.
Refinement. Hydrogen atoms attached to carbon atoms were placed at calculated positions and were refined in the riding-model approximation with C—H = 0.95–0.99 Å, and with *U*_iso_(H) = 1.2*U*_eq_(C) or 1.5 *U*_eq_(methyl C).

### Methyl 3-{4,4-difluoro-2-[2-(methoxycarbonyl)ethyl]-1,3,5,7-tetramethyl-4-bora-3a,4a-diaza-s-indacen-6-yl}propionate Fractional atomic coordinates and isotropic or equivalent isotropic displacement parameters (Å^2^)

**Table:**

	*x*	*y*	*z*	*U*_iso_*/*U*_eq_	
C1	0.6168 (4)	0.1527 (2)	0.1730 (5)	0.0375 (10)	
C2	0.6242 (4)	0.0886 (2)	0.1758 (5)	0.0401 (10)	
N3A	0.4545 (3)	0.11329 (17)	0.2243 (4)	0.0357 (8)	
C3	0.5240 (4)	0.0654 (2)	0.2098 (5)	0.0365 (10)	
N4A	0.2907 (3)	0.17653 (16)	0.2632 (4)	0.0320 (8)	
B4	0.3319 (5)	0.1102 (2)	0.2480 (6)	0.0345 (10)	
C5	0.1906 (4)	0.1955 (2)	0.2927 (5)	0.0321 (9)	
C6	0.1870 (4)	0.2612 (2)	0.2985 (5)	0.0339 (9)	
C7	0.2882 (4)	0.2830 (2)	0.2691 (5)	0.0334 (9)	
C7A	0.3531 (4)	0.2300 (2)	0.2478 (5)	0.0332 (9)	
C8	0.4593 (4)	0.2254 (2)	0.2161 (5)	0.0378 (10)	
H8	0.499101	0.262106	0.202465	0.045*	
C8A	0.5107 (4)	0.1680 (2)	0.2034 (5)	0.0344 (9)	
F9	0.2522 (2)	0.08108 (12)	0.1056 (3)	0.0419 (6)	
F10	0.3365 (2)	0.07568 (12)	0.3926 (3)	0.0463 (7)	
C11	0.7237 (4)	0.0497 (2)	0.1543 (6)	0.0447 (11)	
H11A	0.765621	0.073310	0.088177	0.054*	
H11B	0.690710	0.011961	0.089141	0.054*	
C12	0.8112 (4)	0.0313 (2)	0.3255 (6)	0.0462 (12)	
H12A	0.845614	0.069219	0.388989	0.055*	
H12B	0.768455	0.009078	0.392899	0.055*	
C13	0.9092 (4)	−0.0090 (2)	0.3081 (6)	0.0421 (11)	
O14	0.9331 (3)	−0.05959 (17)	0.3704 (5)	0.0525 (9)	
O15	0.9673 (3)	0.01777 (16)	0.2139 (4)	0.0482 (8)	
C16	1.0658 (5)	−0.0171 (3)	0.1959 (7)	0.0571 (14)	
H16A	1.112435	0.009131	0.144368	0.086*	
H16B	1.036517	−0.053078	0.122765	0.086*	
H16C	1.115511	−0.031056	0.308257	0.086*	
C17	0.0914 (4)	0.2973 (2)	0.3389 (5)	0.0365 (10)	
H17A	0.120908	0.339517	0.374704	0.044*	
H17B	0.074036	0.277407	0.436257	0.044*	
C18	−0.0232 (4)	0.3022 (2)	0.1904 (5)	0.0343 (9)	
H18A	−0.009617	0.328626	0.100521	0.041*	
H18B	−0.046000	0.260563	0.141415	0.041*	
C19	−0.1220 (4)	0.3287 (2)	0.2435 (5)	0.0372 (10)	
O20	−0.1238 (3)	0.33229 (16)	0.3882 (4)	0.0449 (8)	
O21	−0.2120 (3)	0.34756 (15)	0.1088 (4)	0.0412 (7)	
C22	−0.3121 (4)	0.3733 (3)	0.1478 (7)	0.0512 (13)	
H22A	−0.376353	0.380654	0.041848	0.077*	
H22B	−0.289255	0.412509	0.208816	0.077*	
H22C	−0.338522	0.344308	0.219404	0.077*	
C23	0.7046 (5)	0.1981 (2)	0.1491 (7)	0.0506 (12)	
H23A	0.663003	0.233367	0.082459	0.076*	
H23B	0.753553	0.178161	0.088545	0.076*	
H23C	0.754893	0.212549	0.260557	0.076*	
C24	0.4960 (4)	0.0000 (2)	0.2359 (6)	0.0420 (11)	
H24A	0.523288	−0.026676	0.160100	0.063*	
H24B	0.410510	−0.004618	0.210398	0.063*	
H24C	0.535739	−0.011763	0.354578	0.063*	
C25	0.0994 (4)	0.1511 (2)	0.3080 (6)	0.0399 (10)	
H25A	0.045183	0.172126	0.357789	0.060*	
H25B	0.137629	0.116608	0.381501	0.060*	
H25C	0.055169	0.135340	0.194887	0.060*	
C26	0.3262 (4)	0.3483 (2)	0.2625 (5)	0.0377 (10)	
H26A	0.339927	0.355885	0.153316	0.057*	
H26B	0.399334	0.355734	0.355804	0.057*	
H26C	0.264443	0.376188	0.273969	0.057*	

### Methyl 3-{4,4-difluoro-2-[2-(methoxycarbonyl)ethyl]-1,3,5,7-tetramethyl-4-bora-3a,4a-diaza-s-indacen-6-yl}propionate Atomic displacement parameters (Å^2^)

**Table:**

	*U*^11^	*U*^22^	*U*^33^	*U*^12^	*U*^13^	*U*^23^
C1	0.039 (2)	0.046 (3)	0.031 (2)	0.002 (2)	0.0166 (19)	−0.0004 (19)
C2	0.043 (2)	0.045 (3)	0.032 (2)	0.006 (2)	0.0103 (19)	−0.0036 (19)
N3A	0.040 (2)	0.037 (2)	0.0308 (18)	0.0052 (16)	0.0126 (16)	0.0006 (15)
C3	0.042 (2)	0.038 (2)	0.031 (2)	0.004 (2)	0.0129 (18)	−0.0005 (18)
N4A	0.0363 (19)	0.0325 (19)	0.0314 (18)	0.0011 (15)	0.0164 (15)	0.0021 (14)
B4	0.042 (3)	0.038 (3)	0.025 (2)	0.003 (2)	0.013 (2)	0.0021 (19)
C5	0.035 (2)	0.039 (2)	0.0240 (19)	0.0021 (18)	0.0110 (17)	−0.0012 (16)
C6	0.038 (2)	0.041 (2)	0.0244 (19)	0.0009 (19)	0.0122 (17)	−0.0015 (17)
C7	0.038 (2)	0.037 (2)	0.025 (2)	0.0002 (18)	0.0088 (17)	−0.0012 (16)
C7A	0.039 (2)	0.036 (2)	0.025 (2)	0.0024 (19)	0.0123 (17)	−0.0001 (17)
C8	0.042 (2)	0.041 (3)	0.033 (2)	0.004 (2)	0.0153 (19)	0.0032 (19)
C8A	0.039 (2)	0.034 (2)	0.034 (2)	0.0003 (19)	0.0155 (18)	−0.0020 (17)
F9	0.0437 (14)	0.0432 (15)	0.0382 (14)	−0.0020 (12)	0.0123 (11)	−0.0047 (11)
F10	0.0596 (17)	0.0445 (16)	0.0405 (14)	0.0089 (13)	0.0238 (13)	0.0098 (12)
C11	0.044 (3)	0.050 (3)	0.041 (3)	0.005 (2)	0.014 (2)	−0.009 (2)
C12	0.045 (3)	0.056 (3)	0.038 (2)	0.009 (2)	0.014 (2)	−0.003 (2)
C13	0.036 (2)	0.050 (3)	0.038 (2)	−0.002 (2)	0.0085 (19)	0.002 (2)
O14	0.053 (2)	0.049 (2)	0.053 (2)	0.0036 (17)	0.0130 (17)	0.0062 (17)
O15	0.0476 (19)	0.051 (2)	0.052 (2)	0.0070 (16)	0.0231 (16)	0.0032 (16)
C16	0.053 (3)	0.067 (4)	0.057 (3)	0.012 (3)	0.025 (3)	0.002 (3)
C17	0.041 (2)	0.044 (3)	0.028 (2)	0.005 (2)	0.0146 (18)	−0.0009 (18)
C18	0.037 (2)	0.040 (2)	0.027 (2)	0.0010 (19)	0.0126 (17)	−0.0018 (17)
C19	0.043 (2)	0.039 (2)	0.033 (2)	−0.001 (2)	0.0158 (19)	−0.0009 (18)
O20	0.0460 (18)	0.058 (2)	0.0358 (17)	0.0070 (16)	0.0203 (14)	0.0003 (15)
O21	0.0360 (16)	0.051 (2)	0.0364 (16)	0.0061 (15)	0.0112 (13)	0.0013 (14)
C22	0.037 (2)	0.066 (3)	0.054 (3)	0.011 (2)	0.019 (2)	0.004 (3)
C23	0.050 (3)	0.053 (3)	0.055 (3)	−0.003 (2)	0.025 (2)	−0.003 (2)
C24	0.047 (3)	0.040 (3)	0.039 (2)	0.004 (2)	0.012 (2)	−0.001 (2)
C25	0.042 (2)	0.048 (3)	0.036 (2)	0.000 (2)	0.019 (2)	0.0010 (19)
C26	0.044 (2)	0.037 (2)	0.033 (2)	0.000 (2)	0.0123 (19)	−0.0003 (18)

### Methyl 3-{4,4-difluoro-2-[2-(methoxycarbonyl)ethyl]-1,3,5,7-tetramethyl-4-bora-3a,4a-diaza-s-indacen-6-yl}propionate Geometric parameters (Å, º)

**Table:**

C1—C2	1.389 (6)	C13—O15	1.326 (5)
C1—C8A	1.405 (6)	O15—C16	1.443 (6)
C1—C23	1.494 (6)	C16—H16A	0.9800
C2—C3	1.404 (6)	C16—H16B	0.9800
C2—C11	1.510 (6)	C16—H16C	0.9800
N3A—C3	1.356 (5)	C17—C18	1.528 (6)
N3A—C8A	1.396 (5)	C17—H17A	0.9900
N3A—B4	1.537 (6)	C17—H17B	0.9900
C3—C24	1.483 (6)	C18—C19	1.496 (6)
N4A—C5	1.355 (5)	C18—H18A	0.9900
N4A—C7A	1.403 (5)	C18—H18B	0.9900
N4A—B4	1.535 (6)	C19—O20	1.205 (5)
B4—F10	1.395 (5)	C19—O21	1.345 (5)
B4—F9	1.409 (5)	O21—C22	1.442 (5)
C5—C6	1.424 (6)	C22—H22A	0.9800
C5—C25	1.486 (6)	C22—H22B	0.9800
C6—C7	1.386 (6)	C22—H22C	0.9800
C6—C17	1.504 (6)	C23—H23A	0.9800
C7—C7A	1.424 (6)	C23—H23B	0.9800
C7—C26	1.490 (6)	C23—H23C	0.9800
C7A—C8	1.375 (6)	C24—H24A	0.9800
C8—C8A	1.403 (6)	C24—H24B	0.9800
C8—H8	0.9500	C24—H24C	0.9800
C11—C12	1.525 (7)	C25—H25A	0.9800
C11—H11A	0.9900	C25—H25B	0.9800
C11—H11B	0.9900	C25—H25C	0.9800
C12—C13	1.501 (6)	C26—H26A	0.9800
C12—H12A	0.9900	C26—H26B	0.9800
C12—H12B	0.9900	C26—H26C	0.9800
C13—O14	1.205 (6)		
			
C2—C1—C8A	106.8 (4)	C13—O15—C16	115.1 (4)
C2—C1—C23	127.9 (4)	O15—C16—H16A	109.5
C8A—C1—C23	125.3 (4)	O15—C16—H16B	109.5
C1—C2—C3	107.8 (4)	H16A—C16—H16B	109.5
C1—C2—C11	127.0 (4)	O15—C16—H16C	109.5
C3—C2—C11	125.1 (4)	H16A—C16—H16C	109.5
C3—N3A—C8A	107.8 (3)	H16B—C16—H16C	109.5
C3—N3A—B4	127.6 (4)	C6—C17—C18	114.1 (3)
C8A—N3A—B4	124.4 (4)	C6—C17—H17A	108.7
N3A—C3—C2	109.1 (4)	C18—C17—H17A	108.7
N3A—C3—C24	122.9 (4)	C6—C17—H17B	108.7
C2—C3—C24	127.9 (4)	C18—C17—H17B	108.7
C5—N4A—C7A	106.8 (3)	H17A—C17—H17B	107.6
C5—N4A—B4	128.4 (4)	C19—C18—C17	112.3 (3)
C7A—N4A—B4	124.8 (3)	C19—C18—H18A	109.1
F10—B4—F9	108.2 (4)	C17—C18—H18A	109.1
F10—B4—N4A	111.0 (3)	C19—C18—H18B	109.1
F9—B4—N4A	109.7 (4)	C17—C18—H18B	109.1
F10—B4—N3A	110.4 (4)	H18A—C18—H18B	107.9
F9—B4—N3A	109.4 (3)	O20—C19—O21	123.0 (4)
N4A—B4—N3A	108.2 (4)	O20—C19—C18	125.2 (4)
N4A—C5—C6	110.2 (4)	O21—C19—C18	111.8 (3)
N4A—C5—C25	122.0 (4)	C19—O21—C22	115.7 (3)
C6—C5—C25	127.7 (4)	O21—C22—H22A	109.5
C7—C6—C5	107.2 (4)	O21—C22—H22B	109.5
C7—C6—C17	128.7 (4)	H22A—C22—H22B	109.5
C5—C6—C17	123.9 (4)	O21—C22—H22C	109.5
C6—C7—C7A	106.6 (4)	H22A—C22—H22C	109.5
C6—C7—C26	128.3 (4)	H22B—C22—H22C	109.5
C7A—C7—C26	125.0 (4)	C1—C23—H23A	109.5
C8—C7A—N4A	120.2 (4)	C1—C23—H23B	109.5
C8—C7A—C7	130.7 (4)	H23A—C23—H23B	109.5
N4A—C7A—C7	109.1 (3)	C1—C23—H23C	109.5
C7A—C8—C8A	121.9 (4)	H23A—C23—H23C	109.5
C7A—C8—H8	119.0	H23B—C23—H23C	109.5
C8A—C8—H8	119.0	C3—C24—H24A	109.5
N3A—C8A—C8	120.2 (4)	C3—C24—H24B	109.5
N3A—C8A—C1	108.5 (4)	H24A—C24—H24B	109.5
C8—C8A—C1	131.3 (4)	C3—C24—H24C	109.5
C2—C11—C12	111.8 (4)	H24A—C24—H24C	109.5
C2—C11—H11A	109.3	H24B—C24—H24C	109.5
C12—C11—H11A	109.3	C5—C25—H25A	109.5
C2—C11—H11B	109.3	C5—C25—H25B	109.5
C12—C11—H11B	109.3	H25A—C25—H25B	109.5
H11A—C11—H11B	107.9	C5—C25—H25C	109.5
C13—C12—C11	112.9 (4)	H25A—C25—H25C	109.5
C13—C12—H12A	109.0	H25B—C25—H25C	109.5
C11—C12—H12A	109.0	C7—C26—H26A	109.5
C13—C12—H12B	109.0	C7—C26—H26B	109.5
C11—C12—H12B	109.0	H26A—C26—H26B	109.5
H12A—C12—H12B	107.8	C7—C26—H26C	109.5
O14—C13—O15	123.4 (4)	H26A—C26—H26C	109.5
O14—C13—C12	125.0 (4)	H26B—C26—H26C	109.5
O15—C13—C12	111.6 (4)		
			
C8A—C1—C2—C3	−0.8 (5)	C17—C6—C7—C26	−2.9 (7)
C23—C1—C2—C3	177.2 (4)	C5—N4A—C7A—C8	−179.6 (4)
C8A—C1—C2—C11	−178.2 (4)	B4—N4A—C7A—C8	0.2 (6)
C23—C1—C2—C11	−0.2 (8)	C5—N4A—C7A—C7	0.1 (4)
C8A—N3A—C3—C2	−1.3 (5)	B4—N4A—C7A—C7	180.0 (4)
B4—N3A—C3—C2	175.1 (4)	C6—C7—C7A—C8	−179.9 (4)
C8A—N3A—C3—C24	176.0 (4)	C26—C7—C7A—C8	−0.7 (7)
B4—N3A—C3—C24	−7.6 (7)	C6—C7—C7A—N4A	0.4 (4)
C1—C2—C3—N3A	1.3 (5)	C26—C7—C7A—N4A	179.6 (4)
C11—C2—C3—N3A	178.8 (4)	N4A—C7A—C8—C8A	−2.0 (6)
C1—C2—C3—C24	−175.8 (4)	C7—C7A—C8—C8A	178.3 (4)
C11—C2—C3—C24	1.7 (7)	C3—N3A—C8A—C8	−178.6 (4)
C5—N4A—B4—F10	−55.6 (6)	B4—N3A—C8A—C8	4.9 (6)
C7A—N4A—B4—F10	124.6 (4)	C3—N3A—C8A—C1	0.8 (5)
C5—N4A—B4—F9	63.9 (5)	B4—N3A—C8A—C1	−175.8 (4)
C7A—N4A—B4—F9	−116.0 (4)	C7A—C8—C8A—N3A	−0.5 (6)
C5—N4A—B4—N3A	−176.9 (4)	C7A—C8—C8A—C1	−179.7 (4)
C7A—N4A—B4—N3A	3.3 (5)	C2—C1—C8A—N3A	0.0 (5)
C3—N3A—B4—F10	56.7 (6)	C23—C1—C8A—N3A	−178.1 (4)
C8A—N3A—B4—F10	−127.4 (4)	C2—C1—C8A—C8	179.3 (5)
C3—N3A—B4—F9	−62.3 (5)	C23—C1—C8A—C8	1.2 (8)
C8A—N3A—B4—F9	113.6 (4)	C1—C2—C11—C12	95.7 (6)
C3—N3A—B4—N4A	178.3 (4)	C3—C2—C11—C12	−81.3 (6)
C8A—N3A—B4—N4A	−5.8 (5)	C2—C11—C12—C13	178.2 (4)
C7A—N4A—C5—C6	−0.6 (5)	C11—C12—C13—O14	−123.1 (5)
B4—N4A—C5—C6	179.6 (4)	C11—C12—C13—O15	56.6 (6)
C7A—N4A—C5—C25	177.1 (4)	O14—C13—O15—C16	−2.4 (7)
B4—N4A—C5—C25	−2.8 (6)	C12—C13—O15—C16	177.9 (4)
N4A—C5—C6—C7	0.8 (5)	C7—C6—C17—C18	105.4 (5)
C25—C5—C6—C7	−176.6 (4)	C5—C6—C17—C18	−78.1 (5)
N4A—C5—C6—C17	−176.4 (3)	C6—C17—C18—C19	170.2 (4)
C25—C5—C6—C17	6.2 (7)	C17—C18—C19—O20	−17.2 (6)
C5—C6—C7—C7A	−0.7 (5)	C17—C18—C19—O21	164.1 (4)
C17—C6—C7—C7A	176.3 (4)	O20—C19—O21—C22	0.7 (7)
C5—C6—C7—C26	−179.8 (4)	C18—C19—O21—C22	179.4 (4)

### Methyl 3-{4,4-difluoro-2-[2-(methoxycarbonyl)ethyl]-1,3,5,7-tetramethyl-4-bora-3a,4a-diaza-s-indacen-6-yl}propionate Hydrogen-bond geometry (Å, º)

**Table:**

*D*—H···*A*	*D*—H	H···*A*	*D*···*A*	*D*—H···*A*
C16—H16*A*···F9^i^	0.98	2.37	3.324 (7)	163
C18—H18*A*···O14^ii^	0.99	2.57	3.268 (6)	128
C23—H23*B*···O20^iii^	0.98	2.54	3.470 (7)	158

Symmetry codes: (i) *x*+1, *y*, *z*; (ii) −*x*+1, *y*+1/2, −*z*+1/2; (iii) *x*+1, −*y*+1/2, *z*−1/2.
